# Sternocostoclavicular Hyperostosis: An Ill-Recognized Disease

**DOI:** 10.3390/diagnostics6030029

**Published:** 2016-08-05

**Authors:** Bolette Roed, Tatiana Kristensen, Søren Thorsen, Klaus Poulsen Bloch, Pia Afzelius

**Affiliations:** 1 Department of Diagnostic Imaging, Nordsjællands Hospital Hillerød, Copenhagen University Hospital, Dyrehavevej 29, 3400 Hillerød, Denmark; tatiana.kristensen.01@regionh.dk (T.K.); klaus.poulsen.bloch@regionh.dk (K.P.B.); pia.maria.tullia.afzelius@regionh.dk (P.A.); 2 Department of Radiology, Rigshospitalet, Copenhagen University Hospital Copenhagen, Blegdamsvej 9, 2100 København Ø, Denmark; 3 Department of Pulmonary and Infectious Diseases, Nordsjællands Hospital Hillerød, Copenhagen University Hospital, Dyrehavevej 29, 3400 Hillerød, Denmark; soeren.thorsen@regionh.dk

**Keywords:** sternocostoclavicular hyperostosis, SCCH, sternocostoclavicular joint, shoulder girdle, bullhead sign, technetium-99m diphosphonate, SAPHO syndrome

## Abstract

Sternocostoclavicular hyperostosis (SCCH) is an ill-recognized, rarely diagnosed disease. Today, SCCH is widely considered part of the synovitis, acne, pustulosis, hyperostosis and osteitis (SAPHO) syndrome. SCCH develops over years with intermittent attacks of pain, swelling, and reddening of the sternocostoclavicular region. The disease causes progressive hyperostosis, fusion of the sternocostoclavicular joints, and soft tissue ossification. SCCH is chronic, non-malignant, and occurs predominantly bilaterally in middle-aged women. The incidence of the disease is unknown. We present a case of isolated SCCH, where chest radiographs showed a clear development of bilateral disease over the course of more than a decade. Whole-body bone scintigraphy was performed and was suggestive of SCCH. The diagnosis was established as late as 14 years from the onset of symptoms. During this period, the patient underwent several inconclusive examinations, resulting in a delay of diagnosis and in prolonged and aggravated symptoms. With this case report, we want to draw attention to SCCH and the importance of early diagnosis of the disease.

**Figure 1:**
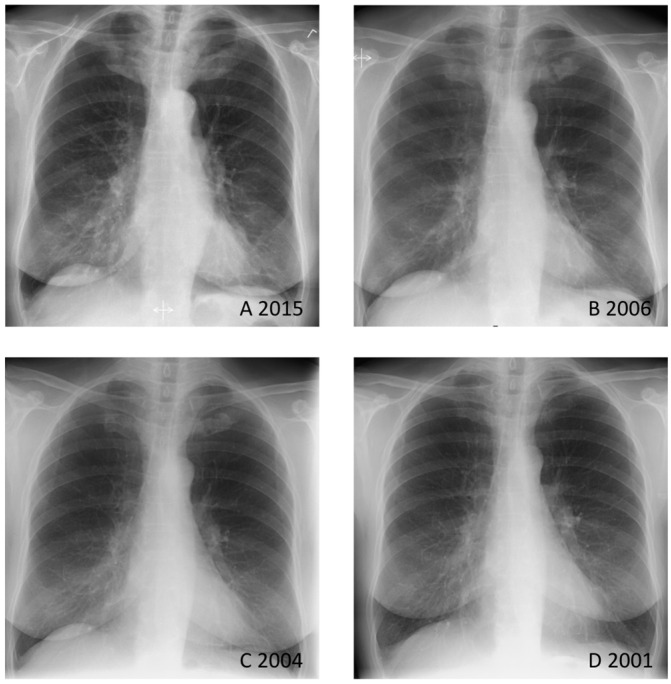
The patient was a healthy 73-year-old woman suffering from intermittent pain in the upper part of the sternum over several years. A chest radiograph from 2015 showed marked bilateral sternocostoclavicular hyperostosis (SCCH) (**A**); Retrospectively, chest radiographs from 2006 (**B**) and 2004 (**C**) revealed that sternocostoclavicular sclerotic changes had already begun—especially when compared with a chest radiograph from 2001 (**D**). Sternocostoclavicular hyperostosis (SCCH) was described for the first time in Europe in 1975 [1]. Shortly thereafter, in 1977, two new patients presented with the same symptoms of painful swelling of the sternum, clavicles, and upper ribs [2]. In the years to come SCCH was observed in patients in association with various skin lesions, and in 1987 the acronym SAPHO (synovitis, acne, pustulosis, hyperostosis and osteitis) was proposed [3]. Today, SCCH is widely considered to be part of the SAPHO syndrome. SAPHO describes the association between osteoarticular and dermatological lesions, and SCCH is only one of many entities which constitutes SAPHO [4,5,6]. In this patient case, no skin lesions were observed, suggesting SCCH as an isolated entity.

**Figure 2:**
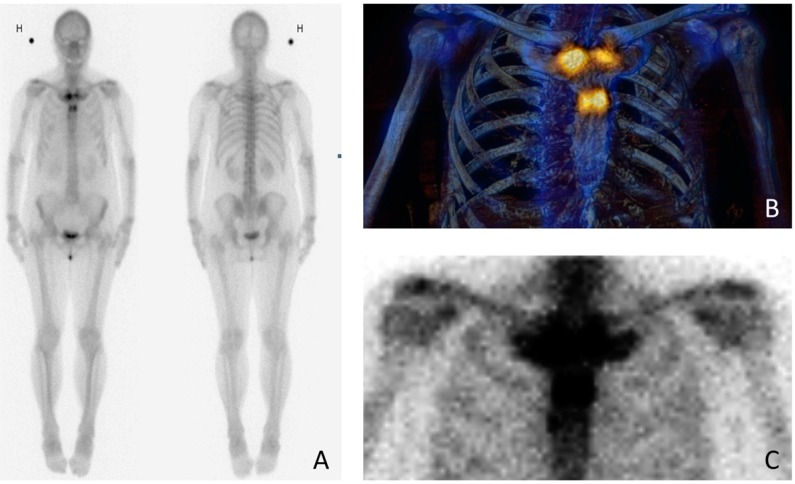
To rule out malignancy, the patient was examined with whole-body bone scintigraphy with both planar (**A**) and single photon emission computed tomography (SPECT) (**B**)/low-dose computed tomography (CT) images (**C**). Scintigraphy was performed in 2015, within two weeks of the chest radiograph (Figure 1A). The images demonstrate abnormal radiotracer activity in both sternocostoclavicular joints, in the sternal angle and manubrium and also in the proximal body of the sternum. The symmetric tracer activity adjacent to the joints rules out malignancy; instead, scintigraphy shows a typical “bullhead sign” [7]. The bullhead sign consists of increased scintigraphic activity in the sternal manubrium and in the adjacent clavicles and ribs, representing the skull and horns of the bull, respectively. The increased scintigraphic activity corresponds to the radiographic changes caused by the hyperostosis. The bullhead sign is a characteristic scintigraphic pattern in demonstrating SCCH [7,8,9]. H stands for højre (Danish) = right side of the patient. The possibilities of diagnostic imaging are wide. Chest radiographs, Tc-99 m bone scintigraphy, computed tomography (CT) or magnetic resonance imaging (MRI) are all used to various extent [4,5,7,10]. Whole-body bone scintigraphy and MRI (including T1-weighted (T1W), short tau inversion recovery (STIR) with or without T1W post-gadolinium sequences) have both proven to be of great importance where radiographically occult sites hamper the diagnosis [5,7]. In case of extensive follow-up imaging, MRI has the advantage of being radiation-free.

**Figure 3:**
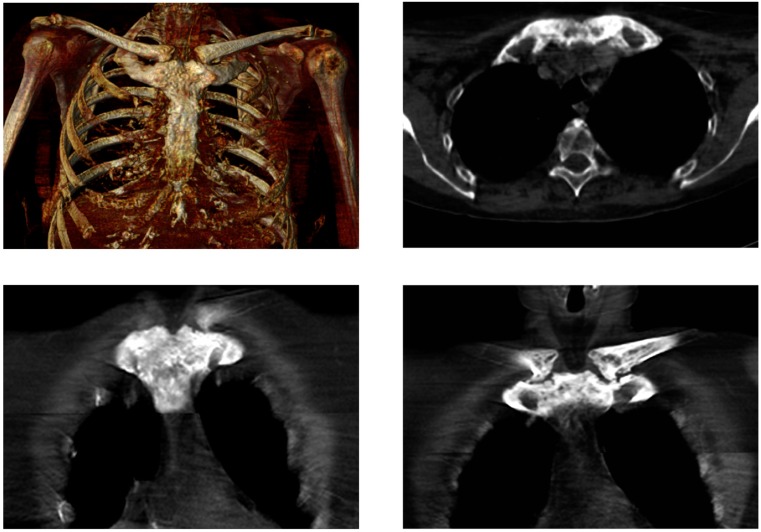
Low-dose CT, in line with bone scintigraphy (Figure 2, 2015) shows symmetrical hyperostosis of the sternal manubrium, medial parts of both clavicles, and anterior parts of the uppermost ribs. SCCH is a rare disorder typically starting with nonspecific inflammation of the sternocostoclavicular ligaments and the surrounding soft tissue. The disease is benign but chronic, and develops over several years, with progressive hyperostosis, fusion of the sternocostoclavicular joints, and soft tissue ossification. These findings are associated with intermittent attacks of pain, swelling, and reddening of the region. Most often the disease is bilateral. The pain may extend to the neck and shoulder, and the mobility of the shoulder and arm may be restricted. The incidence of SCCH is not known, partly because the current literature consists of case reports only, and partly because the condition is underdiagnosed. SCCH is a disease predominantly seen in middle-aged women. Despite its characteristic clinical features, it is an ill-recognized disease with a low level of awareness often leading to a delay in diagnosis. Characteristically, it takes a median of 3.5 years from the patient’s first consultation at the general practitioner until the establishment of the diagnosis. As SCCH is a chronic disease, treatment is mainly aimed at pain relief [9,11,12]. In conclusion, we would like to draw attention to the importance of the early diagnosis of SCCH. We furthermore want to stress that in patients with SCCH, the bullhead sign allows for an early diagnosis, being a characteristic scintigraphic pattern.

## References

[1] Köhler H., Uehlinger E., Kutzner J., Weihrauch T.R., Wilbert L., Schuster R. (1975). Sternocosto-clavicular hyperostosis: A hitherto undescribed entity. Dtsch. Med. Wochenschr..

[2] Köhler H., Uehlinger E., Kutzner J., West T.B. (1977). Sternocostoclavicular hyperostosis: Painful swelling of the sternum, clavicles, and upper ribs. Ann. Intern. Med..

[3] Chamot A.M., Benhamou C.L., Kahn M.F., Beraneck L., Kaplan G., Prost A. (1987). Acne-pustulosis-hyperostosis-osteitis syndrome. Results of a national survey 85 cases. Rev. Rhum. Mal. Ostéoartic..

[4] Heldmann F., Kiltz U., Baraliakos X., Braun J. (2014). SAPHO syndrome. Z. Rheum..

[5] Depasquale R., Kumar N., Lalam R.K., Tins B.J., Tyrrell P.N.M., Singh J., Cassar-Pullicino V.N. (2012). SAPHO: What radiologists should know. Clin. Radiol..

[6] Köhler H. (2013). From Sternocostoclavicular Hyperostosis (SCCH) to SAPHO syndrome. Curr. Rheumatol. Rev..

[7] Freyschmidt J., Sternberg A. (1998). The bullhead sign: Scintigraphic pattern of sternocostoclavicular hyperostosis and pustulotic arthroosteitis. Eur. Radiol..

[8] Kim B.Y., Karak P., Bybel B., Freedman G.S., Neumann D.R. (2001). Sternocostoclavicular hyperostosis. scintigraphic evaluation. Clin. Nucl. Med..

[9] Caroll M.B. (2011). Sternocostoclavicular hyperostosis: a review. Ther. Adv. Musculoskelt. Dis..

[10] Fritz P., Baldauf G., Wilke H.-J., Reitter I. (1992). Sternocostoclavicular hyperostosis: Its progression and radiological features. A study of 12 cases. Ann. Rheum. Dis..

[11] Kloot W.A., Chotkan S.A., Kaptein A.A., Hamidy N.A.T. (2010). Diagnostic delay in sternocostoclavicular hyperostosis: Impact on various aspects of quality of life. Arthritis Care Res..

[12] Nguyen M.T., Borchers A., Selmi C., Naguwa S.M., Cheema G., Gershwin E. (2012). The SAPHO Syndrome. Semin. Arthritis Rheum..

